# Molecular Mechanisms Related to Responses to Oxidative Stress and Antioxidative Therapies in COVID-19: A Systematic Review

**DOI:** 10.3390/antiox11081609

**Published:** 2022-08-19

**Authors:** Evangelia Eirini Tsermpini, Una Glamočlija, Fulden Ulucan-Karnak, Sara Redenšek Trampuž, Vita Dolžan

**Affiliations:** 1 Pharmacogenetics Laboratory, Institute of Biochemistry and Molecular Genetics, Faculty of Medicine, University of Ljubljana, 1000 Ljubljana, Slovenia; 2 Faculty of Pharmacy, University of Sarajevo, 71000 Sarajevo, Bosnia and Herzegovina; 3 School of Medicine, University of Mostar, 88000 Mostar, Bosnia and Herzegovina; 4 Department of Medical Biochemistry, Faculty of Medicine, Ege University, Bornova, 35100 İzmir, Turkey

**Keywords:** COVID-19, oxidative stress, antioxidant enzymes, genetics, anti-oxidative therapies, glutathione, N-acetylcysteine

## Abstract

The coronavirus disease (COVID-19) pandemic is a leading global health and economic challenge. What defines the disease’s progression is not entirely understood, but there are strong indications that oxidative stress and the defense against reactive oxygen species are crucial players. A big influx of immune cells to the site of infection is marked by the increase in reactive oxygen and nitrogen species. Our article aims to highlight the critical role of oxidative stress in the emergence and severity of COVID-19 and, more importantly, to shed light on the underlying molecular and genetic mechanisms. We have reviewed the available literature and clinical trials to extract the relevant genetic variants within the oxidative stress pathway associated with COVID-19 and the anti-oxidative therapies currently evaluated in the clinical trials for COVID-19 treatment, in particular clinical trials on glutathione and N-acetylcysteine.

## 1. Introduction

Coronavirus disease (COVID-19) is the disease caused by a new coronavirus called severe acute respiratory syndrome coronavirus 2 (SARS-CoV-2), originated from Wuhan, China [1]. The COVID-19 pandemic is a leading global health and economic challenge and is associated with high mortality and morbidity rates [2,3,4]. However, the new Omicron strains are associated with higher infectivity and lower mortality (https://www.who.int/en/activities/tracking-SARS-CoV-2-variants/; accessed on 12 July 2022). According to the World Health Organization (WHO), since the COVID-19 outbreak and until the 15th of June 2022, over 409 million confirmed cases and around 6 million deaths had been reported globally (https://covid19.who.int/?; accessed on 15 July 2022). Regarding the severity of its symptoms, COVID-19 can be mild, moderate, severe, or critical [5], but also asymptomatic cases have been reported, which contribute to the spreading of the virus [6]. Patients with mild to moderate symptoms experience dyspnea, fatigue, cough, fever, and others. In contrast, those with severe COVID-19 complications might experience sedation, coagulopathy, acute kidney and myocardial injury, renal failure, and others [7,8].

**Figure 1:**
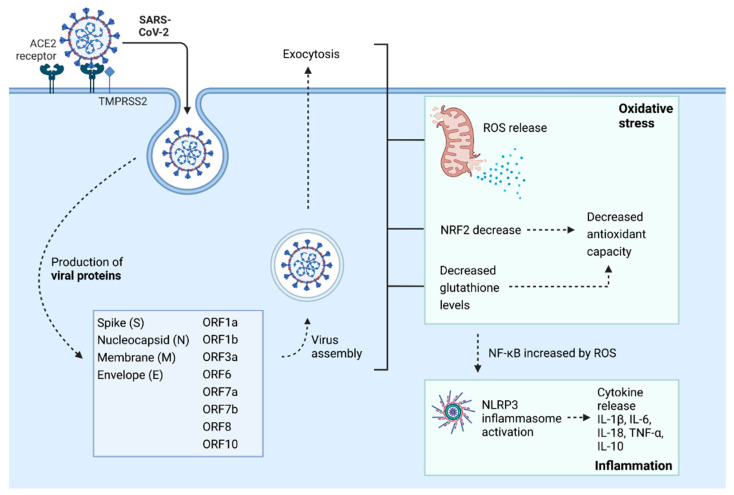
COVID-19 and oxidative stress. Abbreviations: ACE2: Angiotensin-Converting Enzyme 2, SARS-CoV2: severe acute respiratory syndrome coronavirus 2, TMPRSS2: Transmembrane protease, serine 2, ORF1a: open reading frame 1 alpha, ORF1b: open reading frame 1 beta, ORF3a: open reading frame 3 alpha, ORF6: open reading frame 6, ORF7a: open reading frame 7 alpha, ORF7b: open reading frame 7 beta, ORF8: open reading frame 8, ORF10: open reading frame 10, ROS: reactive oxygen species, NRF2: Nuclear factor erythroid 2-related factor 2, NF-kβ: Nuclear factor kappa B, NLRP3: nucleotide-binding domain (NOD)-like receptor (NLR) family pyrin domain containing 3, IL-1β: Interleukin 1 beta, IL-6: Interleukin 6, IL-18: Interleukin 18, IL-10: Interleukin 10, TNF-α: tumor necrosis factor-alpha. Created with BioRender.com (accessed on 9 August 2022).

Factors like age, sex, and concomitant diseases are associated with COVID-19 severity and mortality [9,10,11,12]. However, what defines the disease’s progression is not entirely understood. Still, there are strong indications that oxidative stress and the defense against reactive oxygen species (ROS) are crucial in COVID-19 pathogenesis on various mechanistic levels [13]. Upon the binding of SARS-CoV-2 Spike (S) protein to the ACE2 receptor on the cell membrane, the virus enters the cell by endocytosis, leading to RNA replication and translation of viral structural proteins, such as Spike (S), Nucleocapsid (N), Membrane (M), Envelope (E), as well as viral open reading frames (ORFs) and activation of oxidative stress and inflammatory pathways. ROS are released, and NRF2 and glutathione levels are decreased, leading to decreased antioxidant capacity. At the same time, NF-kB levels are increased by ROS and activate the NLRP3 inflammasome, leading to cytokine activation and inflammation (Figure 1).

Oxidative stress results from the imbalance between ROS production and the cell’s antioxidant capacity [14]. ROS disrupts cellular biochemical pathways by causing DNA strand breaks, lipid peroxidation, and protein modification and degradation [15]. Antioxidant enzymes such as superoxide dismutase (SOD), catalase (CAT), and glutathione peroxidase (GPX) represent a vital defense mechanism against ROS [16]. SODs are metalloenzymes that convert the O_2_^•^ into hydrogen peroxide (H_2_O_2_) and O_2_ [17]. Then, H_2_O_2_ may be further converted into water via CAT and GPX [16,18] or may further contribute to oxidative-stress damage through other biological reactions [17]. CAT is a heme-containing enzyme in the peroxisomes of nearly all aerobic cells [19]. GPX is a substrate-specific enzyme requiring glutathione (GSH), which is one of the essential thiol groups containing antioxidants [20] in our body [21]. Serum concentrations of SOD and GPX were significantly higher in COVID-19 patients [22].

**Figure 2:**
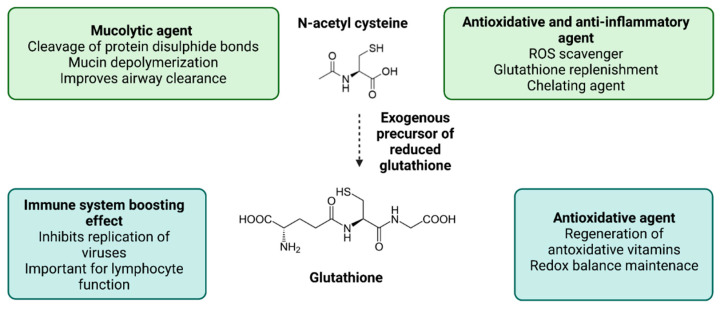
Glutathione and N-acetylcysteine as antioxidant and anti-inflammatory agents. Abbreviations: ROS: reactive oxygen species. Created with BioRender.com (accessed on 9 August 2022).

The imbalance between oxidative stress promoting mechanisms and the defense and repair mechanisms can lead to molecular and cellular damage and trigger the activation of stress response and inflammatory pathways. Therefore, treating patients to prevent or diminish those effects is highly important. Various therapeutic approaches that reduce oxidative stress and are based on antioxidative and anti-inflammatory agents may benefit COVID-19 patients. Since these strategies were used to overcome septic shock and were also successfully applied in other conditions such as acute respiratory distress syndrome and acute lung injury to improve oxygenation rates and glutathione levels and strengthen the immune response, a computational study suggested that they could also be used in COVID-19 [23]. N-Acetylcysteine (NAC) is a derivative of the amino acid L-cysteine and, as such, a thiol compound with a dual antioxidant mechanism. It may directly neutralize free radicals, and it may also act as a donor of cysteine for endogenous GSH production. NAC restores the physiological pool of GSH, exerts direct and indirect antioxidant activity and anti-inflammatory activity, and improves immune T-cell response (Figure 2) [24]. Due to their antioxidant and anti-inflammatory properties, NAC and GSH can be promising interventions to reduce the COVID-19 risk [25]. Studies have also shown that high doses of NAC may prevent the severe symptoms of COVID-19 [26,27,28].

Our article aims to highlight the critical role of oxidative stress in the pathogenesis and severity of COVID-19 and to shed light on the underlying molecular mechanisms. We have also systematically reviewed published findings on the role of oxidative stress levels, antioxidative enzymes, genetic, genomic, and transcriptomic factors affecting their activity, oxidative stress markers, and antioxidant capacity in COVID-19, as well as the published studies and clinical trials on the potential antioxidative therapies in COVID-19. It should be noted that since the most suggested potential therapeutic candidates for COVID-19 are GSH and NAC, this review is mainly focused on these two antioxidant agents.

## 2. Methods

To identify the role of oxidative stress in COVID-19, we systematically searched the PubMed database, using the term (COVID-19 or SARS-CoV-2) and (“oxidative stress”).

We have narrowed the search of the PubMed database by adding the terms (genetics or genomics or genes or polymorphism or “genetic variations”) to identify the genetic association studies and by adding the keyword (transcriptomics) to identify transcriptomic studies focusing on genetic polymorphisms in antioxidative enzymes and oxidative stress-related pathways in COVID-19.

We have also searched the GWAS catalog for GWAS studies in COVID-19 patients. We have identified 11 studies and have included them in the pathway enrichment analysis, as described in details in the Supplementary Material File S1.

Regarding the antioxidative therapies currently evaluated in the clinical trials for COVID-19 treatment, we systematically searched the PubMed database and the clinicaltrials.gov website. For the PubMed search, we used the keywords (SARS-CoV-2 OR COVID-19) (n-acetylcysteine OR NAC) (ther* OR treat*) and (SARS-CoV-2 OR COVID-19) (glutathione OR GSH) (ther* OR treat*). For the clinical trials, we searched on condition or disease as COVID-19 with keywords “N-Acetylcysteine” and “Glutathione.”

More information about the search strategies and the records retrieved can be found in the Supplementary Material File S1.

**Figure 3:**
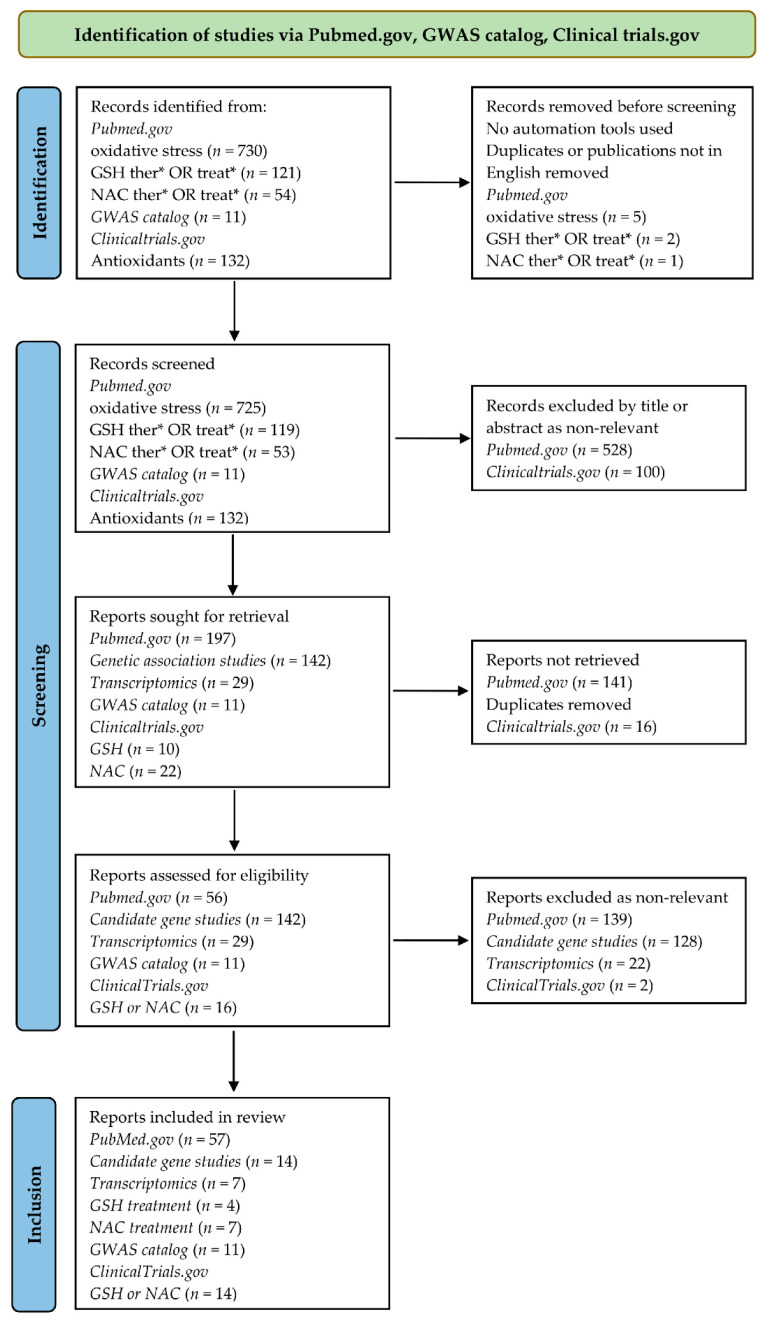
PRISMA diagram. *- asterisk at the root of the PubMed search terms represents any group of characters, including no character.

The Preferred Reporting Items for the Systematic Review and Meta-analysis (PRISMA) statement were used as the reference standard [29]. A PRISMA flow diagram of the systematic search in this review, following Page et al. [29] is shown in Figure 3.

## 3. The Role of Oxidative Stress in COVID-19 Pathogenesis

Several studies found that oxidative stress markers are higher in COVID-19 patients than in healthy subjects [30,31,32] and more severe forms of COVID-19 compared to mild conditions and healthy controls [33]. Although ROS generation is a standard process in the organism with no negative effects while controlled, this balance is disrupted in COVID-19. Mechanisms for the high production of ROS are activated [34,35], while antioxidant mechanisms of cells are reduced [34]. Generally, high ROS production is often seen in respiratory viral infections [36]. Generation of ROS has an essential role in pathology, lifecycle, and/or establishment of RNA viruses such as rhinoviruses [37], neuropathogenic retrovirus ts1 [38], influenza viruses [39], hepatitis C, and human immunodeficiency virus (HIV) [40]. In experiments on mice, SARS-CoV-2 increased ROS production and induced apoptosis in plasma cells producing antibodies against the virus [35]. Moreover, oxidative stress induced the activity of 3CLpro, a key proteinase for SARS-CoV-2 replication [41], interacting with the SARS-CoV-2 nucleocapsid protein involved in the viral life cycle [42]. Twelve SARS-CoV-2 proteins were found to induce oxidative stress, affect proliferation and induce cell death in the yeast cell-based system. SARS-CoV-2 ORF3a protein induced oxidative stress, inflammatory response, and cell death in human pulmonary and kidney epithelial cell lines [43]. Oxidative stress was also induced by SARS-CoV-2 spike protein in human microglial cells [44], THP-1 macrophages, peripheral blood mononuclear cells (PBMCs) [45], and primary bovine aortic endothelial cells [46].

Oxidative stress is often associated with mitochondrial dysfunction [44] and apoptosis [45]. Mitochondria are the primary sources of ROS in cells and are also involved in pathological inflammatory processes and programmed cell death in COVID-19 [47]. Activation of the inflammasomes, loss of mitochondrial membrane potential, and metabolic shift from OXPHOS to glycolysis were observed in monocytes from COVID-19 patients with different disease severity. These changes were observed after short-term recovery, independent of the disease severity [30]. Reduced mitochondrial function in platelets and reduced concentration of coenzyme Q10 in blood and platelets were observed four to seven weeks after COVID-19 infection, both in mild and moderate COVID-19 patients, compared to healthy individuals [48]., Mitochondrial structure and function changes that led to nitrosative stress were observed in postmortem samples from COVID-19 patients with fatal outcomes [49]. Obesity is a risk factor for poor outcomes of COVID-19. Using bioinformatics, Khitan et al. found that oxidative stress due to deregulated Na^+^/K^+^-ATPase transporter signaling could lead to increased risk for more severe forms of the disease in obese individuals [50].

Neutrophils are involved in COVID-19-related oxidative stress. After activation, they produce high levels of ROS as part of their defense mechanisms against pathogens and other targets that need to be destroyed [51,52]. Elevated levels of neutrophils are characteristic of severe COVID-19 patients [30], and a high neutrophil to lymphocyte ratio (NLR) was a predictor of poor prognosis from the early stage of the disease [53]. High NLR was associated with very high levels of ROS production, leading to tissue damage, thrombotic complications, and further development of disease severity [34,54]. NADPH oxidases (NOXs) are enzymes responsible for neutrophils’ ROS production capacity. Mutations that decrease NOS activity increase susceptibility to infections [51], while the increased activity of NOXs in neutrophils causes inflammation and surrounding tissue damage [55]. There are six family members of NOX enzymes, each involved in ROS production. Their hyperactivation is part of several comorbidities related to poor COVID-19 outcomes, such as diabetes, cardiovascular diseases, and obesity [56]. NOX type 2 (NOX2) is involved in superoxide anion production [57]. Activation of this enzyme was observed in hospitalized COVID-19 patients, and this activation was more pronounced in severe forms admitted to the intensive care unit (ICU). Levels of soluble NOX2-derived peptide, an indicator of NOX2 activation, were significantly higher in COVID-19 hospitalized patients than in controls and ICU COVID-19 patients compared to non-ICU patients [58]. Inhibitors of this enzyme are used to treat inflammatory disorders related to uncontrolled neutrophil activity [57].

In healthy cells, increased levels of ROS induce activation of Nrf2 [59]. Nrf2 is a transcription factor promoting the production of antioxidant enzymes and inflammation resolution. It is negatively regulated by Kelch-like ECH-associated protein 1 (KEAP1) inactivated by ROS [60]. Nrf2 has a crucial role in maintaining the respiratory tract’s homeostasis, and this factor’s dysfunction may lead to various respiratory diseases [61]. Different viruses, including SARS-CoV-2, inhibit Nrf2. In COVID-19 patients’ biopsies, decreased levels of antioxidant proteins controlled by Nrf2 were found. Moreover, infection of Vero hTMPRSS2 cells with SARS-CoV-2 decreased levels of Heme Oxygenase 1 (HO-1) and NAD(P)H quinone oxidoreductase 1 (NQO1) induced by Nrf2 [62]. In children with COVID-19, decreased levels of Nrf2 with reduced total antioxidant status and increased total oxidant status and oxidative stress index were observed [63]. Increased levels of ROS and H_2_O_2_ trigger inflammasome as a significant player in cytokine storm-related to severe forms of COVID-19. Pathological findings of hematological parameters, hypoxia of cells, and cardiovascular complications were also associated with elevated levels of ROS in COVID-19 [64]. Increased inflammasome complex formation in monocytes and increased plasma levels of interleukin-18 (IL-18) was observed in COVID-19 patients with more severe symptoms. Peripheral blood mononuclear cells (PBMCs) from COVID-19 patients released IL-1beta, IL-6, and TNF-alpha ex vivo. Inhibition of NLRP3 inflammasome in these cells led to the decreased production of cytokines [30]. Cytokine levels were positively correlated with oxidative stress markers in COVID-19 patients [65]. Case-control studies related to oxidative stress in COVID-19 are summarized in Table 1.

## 4. Oxidative Stress and COVID-19 Severity

Levels of oxidative stress depend on COVID-19 severity [30] and change during the disease [82], so both factors should be considered when comparing results from different studies. Levels of superoxide anion increased substantially with increased disease severity, while hydrogen peroxide levels were similar in all hospitalized patients, independent of disease severity [83]. Mete et al. found that nitric oxide levels were significantly higher in COVID-19 patients at the moment of hospitalization than in controls [69]. In contrast, Cekerevac et al. found that the nitric oxide levels were lower in severe compared to mild and moderate COVID-19 [83]. Yaghoubi et al. found no significant difference in nitric oxide levels in mild and severe COVID-19 patients and healthy controls, but decreased levels were observed with increased COVID-19 severity [84].

Mehri et al. found that total oxidant status was significantly higher in COVID-19 patients at hospital admission than in healthy controls, independent of their later admission to ICU [18]. The prooxidant-antioxidant ratio was also significantly increased in COVID-19 hospitalized patients compared to healthy controls [72]. Increased oxidative stress compared to healthy controls was observed in various disease severities [30], from mild [71] to moderate and severe COVID-19 patients [48]. Lower levels of vitamin D, higher levels of peroxides in plasma, and higher oxidative stress index were found in severe compared to moderate COVID-19 hospitalized patients [73]. Moreover, significantly higher total oxidant status and oxidative stress index were observed in ICU compared to non-ICU patients [85]. Nevertheless, some studies found no correlation between oxidative stress markers’ levels at admission to ICU and COVID-19 outcome [86]. Zendelovska et al. found a similar oxidative stress index at admission in severe patients independent of outcome. However, depending on the outcome, oxidative stress levels changed between admission and seven days after admission. In severe COVID-19 patients with fatal consequences, increased oxidative stress was observed, while in surviving patients, oxidative stress was decreased during this period [82]. Karkhanei et al. found that total oxidant status increased with increased severity of COVID-19 [87]. On the other side, Aykac et al. found that total antioxidant and total oxidant status were similar for both mild, moderate, and severe groups of hospitalized pediatric and adult patients [88]. Although both studies included a similar number of patients, 96 [87] and 86 [88], respectively, it is difficult to compare both studies as they have used different World Health Organization (WHO) interim guidelines for categorization of disease severity, the first from March 2020 [87] and the second from May 2020 [88]. Furthermore, there was insufficient data on inclusion and exclusion criteria for patients in the Aykac et al. paper [88]. Namely, Karkhanei et al. excluded patients receiving antioxidant therapy [87], while Aykac et al. did not mention this criterion [88]. Studies related to oxidative stress that focused on COVID-19 severity are summarized in Table 2.

## 5. Antioxidative Enzymes Studied in COVID-19

The main antioxidative enzymes investigated concerning COVID-19 are SOD, CAT, and GPX. Yaghoubi et al. found no significant difference in serum activities of SOD and CAT in mild and severe COVID-19 patients compared to controls [84]. In contrast, Lage et al. found higher activity of CAT and SOD in plasma of COVID-19 mild, moderate, and severe patients compared to healthy controls [30]. Martin-Fernandez et al. found increased levels of SOD and CAT in the first morning after hospitalization of COVID-19 patients compared to healthy controls [32]. Cekerevac et al. found significantly higher CAT activity in severe COVID-19 compared to moderate and mild forms. However, moderate conditions had significantly lower CAT activity compared to mild forms. SOD activity and reduced GSH were similar in all hospitalized patients, independent of the disease severity [83]. SOD levels were higher in COVID-19 patients, but there was also a difference depending on subsequent admission to ICU. Patients placed in the ICU had significantly higher SOD levels [18]. Mehri et al. found that CAT activity was considerably higher in COVID-19 patients at hospital admission than in healthy controls, independent of their later admission to the ICU [18]. However, studies found decreased levels of CAT in the placentas of pregnant COVID-19 female patients [76] and reduced SOD in the seminal fluid of COVID-19 male patients [75]. Increased DNA oxidative damage and significantly decreased activity of antioxidant enzymes CAT and glutathione synthetase (GSS) were found in placentas of asymptomatic and symptomatic pregnant COVID-19 female patients compared to healthy controls. Moreover, decreased SOD1 and glutathione reductase (GSR) activities were observed without statistical significance [76]. Hajizadeh Maleki and Tartibian analyzed ROS and SOD activity levels in the seminal fluid of hospitalized COVID-19 patients at the baseline and every ten days until sixty days from admission to the hospital. Hospitalized COVID-19 patients had increased ROS levels compared to healthy controls during the entire period of 60 days. The highest ROS levels in COVID-19 patients were at baseline and ten days. ROS levels were significantly lower than baseline but still considerably higher than controls at later time points. SOD activity was significantly lower in COVID-19 patients compared to controls for 60 days, and there was no significant increase in SOD activity during this period [75]. Decreased SOD activity could be regulated through the JAK2/STAT1 pathway and precipitation of pSTAT1 and SOD as observed in mice immunized with SARS-CoV-2 spike protein [35].

GPX requires GSH for its antioxidative activity [16]. GSH is one of the essential antioxidants in our body [21], containing the thiol group [20]. Glucose-6-phosphate dehydrogenase (G6PD) is involved in the production of NADPH which is required to maintain adequate levels of GSH by GSR [97]. Patients with G6PD deficiency were found to have more severe COVID-19 pneumonia with a more extended period of mechanical ventilation [98].

Compounds containing the thiol group interacted with SARS-CoV-2 in vitro, decreasing virus entry into the cell [99]. GSH interacts and, in certain conditions, decreases the main SARS-CoV-2 protease activity [100]. Bioinformatic tools predicted that SARS-CoV-2 main protease targets GPX1, an enzyme involved in neutralizing lipid peroxides and hydrogen peroxide in organisms [101], as well as a glutamate-cysteine ligase, an enzyme involved in GSH synthesis [102]. In a study on 60 hospitalized COVID-19 patients, GSH deficiency was observed compared to healthy controls. This deficiency depended on age and was more pronounced in older persons. Additionally, in COVID-19 patients, increased lipid peroxidation and damage due to oxidative stress were observed [31]. Compared to control samples, significantly decreased GSH levels were observed in post-mortem cerebral cortex samples from COVID-19 patients [78]. Moreover, compared to controls, higher ROS and lower GSH levels were found in post-mortem testis tissue from COVID-19 patients [79]. SARS-CoV-2 also induced oxidative stress-mediated changes in testes and epididymis from post-mortem COVID-19 autopsies compared to controls [80]. GSH levels decreased with the increased severity of COVID-19 [87]. GSH levels were higher in mild than moderate and severe COVID-19 patients [94]. Lage et al. found decreased GSH levels in PBMC lysates from COVID-19 patients compared to healthy controls [30]. Autopsy brain tissues from ten COVID-19 patients were found to have increased oxidative stress with an increased glutathione disulfide/glutathione ratio compared to controls [77].

Another enzyme that may play an essential role in defense against oxidative stress is paraoxonase-1 (PON-1). This enzyme had significantly decreased activity in hospitalized patients generally, and this decrease was more pronounced in hospitalized SARS-CoV-2 positive compared to SARS-CoV-2 negative patients [81].

## 6. Candidate Gene Studies of Oxidative Stress Pathway in COVID-19

Altered antioxidant enzyme activity has been associated with COVID-19 susceptibility and severity. The observed alterations in activity and expression levels of SOD, CAT, and GPX and their associations with ROS and molecular damage levels can be partially explained by the impact of already known functionally important polymorphisms in these genes. Understanding genetic variability and its contribution to COVID pathology is crucial and can lead to disease prevention, prognosis, and therapy. Exploring the genetic variability of patients with COVID-19 may contribute to a better understanding of genetic susceptibility and lead to disease prevention, prognosis, and treatment. However, very few genetic studies with COVID-19 patients that focus on genetic polymorphisms of genes related to oxidative stress have been published so far (Table 3).

The glutathione S-transferases (GSTs) superfamily has been studied the most among other oxidative stress-related genes for their potential association with COVID-19. GSTs catalyze the conjugation of GSH with electrophiles to protect the cell from oxidative damage and participate in the antioxidant defense mechanisms in the lungs [104]. GSTs polymorphisms were associated with susceptibility and severity of COVID-19 [103]. Two candidate gene association studies reported the association of the *GSTT1* and *GSTM1* gene deletions with the COVID-19 outcomes. Both studies consistently showed that the *GSTT1*−/− genotype is associated with a less favorable outcome. The *GSTT1*−/− genotype carriers had higher COVID-19 prevalence and higher fatality and mortality rates due to COVID-19 [107]. Another study showed that the *GSTT1*−/− genotype carriers had an increased chance of severe COVID-19. The *GSTM1*−/− genotype also increased the odds of a more severe disease course [104]. Both studies indicate that an efficient defense against ROS is vital in combating the disease.

*GSTM1* and *GSTT1* gene deletions as well as *GSTP1* rs1138272 and rs1695, *GSTA1* rs3957357, and *GSTM3* rs1332018 polymorphisms have been investigated in a cohort of 207 COVID-19 patients and 252 healthy individuals of Serbian, Caucasian origin. The findings indicated that *GSTM3* rs1332018 and *GSTP1* rs1695 heterozygotes and carriers of the *GSTP1* rs1138272 Val allele had decreased risk of developing COVID-19. Moreover, a borderline association was observed in carriers of the *GSTM3* rs1332018 C allele. After adjustments for age, sex, smoking status, and comorbidities, the association remained significant only for the *GSTP1* rs1695 and *GSTM3* rs1332018 heterozygous carriers [103]. As *GSTM1* and *GSTT1* deletions have been associated with alterations in enzyme activity and risk of pulmonary fibrosis, a severe symptom of COVID-19, it was suggested that they might be used as predictors of COVID-19 morbidity and mortality [108].

When assessing the cumulative effect of the GST genotypes on COVID-19 development, it was reported that individuals with one or up to three risk-associated genotypes had higher odds of developing COVID-19. The higher the number of the associated genotypes, the higher the risk of developing COVID-19. In addition, carriers of three associated risk genotypes experienced more severe symptoms than those with reference genotypes. It is essential to mention that the protective effect of rs1695 has been highlighted in various respiratory diseases, including asthma, and obstructive pulmonary disease, both in genetic association studies and meta-analyses [109,110,111,112,113,114]. The results also align with reports that *GSTP1* rs1695 Val allele frequencies are higher in countries with higher cases and increased COVID-19 mortality [115].

PON1 is a member of the paraoxonase gene family. After its synthesis, the enzyme is secreted into the circulation, binding to high-density lipoprotein (HDL), protecting the HDL proteins against oxidative modification [116]. Two common PON1 functional polymorphisms can alter enzyme activity. More specifically, it can be increased in the presence of rs662 and decreased in rs854560 [117]. The potential association between these polymorphisms and COVID-19 morbidity and mortality was analyzed in data from 48 countries. According to their results, the rs854560 M allele was associated with both the prevalence and mortality of COVID-19. The prevalence of COVID-19 was further related to the number of COVID-19 tests and the human development index (HDI), an indicator of human development. After adjustments for the above confounders, the association remained, indicating that the higher the frequency of the rs854560 T allele, the higher the prevalence and mortality of COVID-19 [117]. Furthermore, the author also emphasized the importance of sex, age, and ethnicity in COVID-19. Mortality rates are higher in males and Caucasians, probably because they have lower PON1 activity levels and higher rs854560 frequencies than females and are negatively correlated with age. However, it should be noted that this is an ecological study and cannot safely conclude that carriers of the rs854560 M allele are at higher risk of COVID-19 infection or that the mortality risk is higher than in carriers of the rs854560 L allele. Moreover, the author concluded that the decreased activity of the enzyme due to the presence of rs854560 is also a risk factor for the mortality of patients with hypertension infected by COVID-19. This agrees with the findings of Delgado et al. regarding the association between decreased activity and the rise of COVID-19 [118].

HO-1 is an essential enzyme in heme catabolism. The heme oxygenase system has a protective, anti-inflammatory, and antioxidant effect [119,120]. A di-nucleotide repeat of GT in the promoter of the heme oxygenase 1 gene (HMOX1) has been suggested to participate in the COVID-19-induced cytokine storm by affecting the transcription of HO-1 to reactive oxygen species [108,120,121,122]. The number of repeats is inversely related to enzyme activity and mRNA levels of HO-1. Additionally, carriers of the long (L) allele have lower expression levels of HO-1 [119,122,123]. This polymorphism is also associated with acute respiratory distress syndrome (ARDS). Furthermore, it is known that individuals with larger GT repeats are more susceptible to cardiovascular diseases, diabetes, and obesity [108,119,124,125,126]. It is also well known that patients with comorbidities like diabetes and obesity experience more severe COVID-19 [108] and higher mortality rates [123]. These patients also have a higher risk of developing complications [123]. Given the above, the severity of COVID-19 could be partially explained by the repeats of GT in the promoter region of the HO-1 [108,123]. However, future studies are needed to verify such associations, in which the ethnic differences should be considered, given that the L allele is more frequent in European and Japanese populations [122]. rs2071746 is another polymorphism of HO-1 that has also been suggested to influence COVID-19 severity, with the T allele regulating the expression of HO-1 [121,122]. The rs2071746 A allele is associated with a low risk of ischemic heart disease and stroke but not with coronary artery disease or lung function [122].

Nitric oxide is essential in COVID-19, as it develops inflammatory stress to prevent pulmonary hypertension [106,127]. Prooxidant eNOS enzyme is encoded by the *NOS3* gene, which is highly polymorphic. rs2070744, rs179983, and a VNTR in the 4^th^ intron of *NOS3* are functional polymorphisms studied individually or as a haplotype in various diseases compared to the reference haplotype consisting of the wild types of these polymorphisms, which is the most common. A review article focused on the potential protective role of eNOS-derived nitric oxide to severe COVID-19 indicated a direct negative correlation between COVID-19 mortality and the percentage of NOS3 WT haplotype. The research group used COVID-19 death per 100 K population information extracted from John Hopkins University Coronavirus Resources Centre, the COVID data tracker of the U.S. Centers for Disease Control and Prevention, and the U.S. Census Bureau data. It combined it with reference data for the WT NOS3 haplotype from the US, Columbia, Brazil, China, South Korea, India, Saudi Arabia, and Spain [127]. Pehlivan et al. investigated the role of *MBL2* and *NOS3* in a cohort of 79 patients with COVID-19 and 100 individuals with a negative test for SARS-CoV-2 antibodies and two negative PCR tests. *MBL2* rs1800450, *NOS3* rs1799983, and a 23bp VNTR in the intron 4 of *NOS3* were analyzed using PCR-RFLP. The results showed no statistically significant difference in the genotypes and alleles between the two groups, neither for rs1799983 nor the VNTR of *NOS3*. However, the genotype and allele frequencies of rs1800450 differ between positive and negative tested individuals. The A allele was higher in COVID-19 patients, whereas the B allele and the AB genotype were higher in individuals with negative tests. NOS enzyme catalyzes superoxide anion production and promotes nitric oxide synthesis [106].

## 7. Genome-Wide Association Studies and Oxidative Stress Pathway in COVID-19

Very few genome-wide association studies (GWAS) published so far have indicated that the patient’s genetic background of the oxidative stress-related pathways affects clinical outcomes in COVID-19.

According to the GWAS catalog [128], eleven GWAS searching for genetic variants affecting COVID-19 susceptibility, COVID-19 severity, and other COVID-19-related phenotypes have been published so far [129,130,131,132,133,134,135,136,137,138,139]. All eleven identified GWAS studies are presented in Table 4. Altogether, they showed that genetic variability of 441 unique genes and genome regions is associated with COVID-19 occurrence or severity. The pathway enrichment analysis results with the DAVID functional annotation tool showed clusters of at least two genes involved in a distinct pathway. We observed no gene clusters directly involved in oxidative stress-related pathways. However, several identified genes play roles in various inflammatory pathways, which are a great source of ROS and are thus indirectly involved in oxidative stress pathways [140]. A comprehensive list of genes identified in the above-listed GWAS is available in Supplementary Table S1. The output of the pathway enrichment analysis for the GO biological process is available in Supplementary Table S2, for KEGG in Supplementary Table S3, and Reactome in Supplementary Table S4.

As none of the studies identified via the GWAS catalog specifically reported oxidative stress-related genes, we manually searched the PubMed database to obtain more information on the published GWAS studies.

A GWAS performed in 2020 included participants of European ancestry with SARS-CoV-2 test results, 676 positive and 1334 negative from the UK Biobank, and investigated COVID-19 susceptibility. The GWAS was performed using the Affymetrix Axiom UK Biobank array or the UK BiLEVE array and indicated that the intronic variant rs286914, located on *EHF* on 11p13, was associated with the positive SARS-CoV-2 test results. More specifically, the rs286914 A allele carriers had an increased risk of positive SARS-CoV-2 test. *EHF* encodes a protein that belongs to an ETS transcription factor subfamily characterized by epithelial-specific expression and plays an essential role in lung inflammation [141].

The study of Qian et al. was based on rs286914, rs11385942, and rs657152 that emerged from the two GWAS studies mentioned above [129,141]. Starting with eQTL and meQTL analyses identified the genes regulated by these genetic variants. Then, they performed protein interaction network analysis, intracellular location analysis, and gene expression correlation analysis for these genes. According to their results, rs286914 is a cis-eQTL of *CAT*, which suggests that *EHF* genetic variants might have a functional role and alter the expression levels of *CAT*. In addition, they observed protein level interactions between CAT and SARS-CoV-2 related S protein, which is encoded by the ACE2 gene. Both CAT and ACE2 are part of the cell membrane and extracellular matrix. The above findings support the authors’ hypothesis that CAT may affect COVID-19 susceptibility. However, future studies are needed to verify this association [142]. It is worth mentioning that there is evidence of the involvement of CAT in respiratory diseases, such as asthma and pulmonary fibrosis [143,144]. Moreover, a study highlights the anti-inflammatory role of CAT through the regulation of cytokine production and its protective function against oxidative damage and inhibition of SARS-CoV-2 replication [145].

## 8. The Transcriptomic Landscape of the Oxidative Stress Pathway in COVID-19

We have identified 29 transcriptomic studies conducted to elucidate whether a differential expression of genes in the oxidative stress-related pathways is associated with the course of COVID-19.

A study in a Greek cohort of 17 asymptomatic and 15 symptomatic COVID-19 patients assessed gene expression in their blood samples. Altogether, 15 genes were found to be differentially expressed. Among them, the ectodermal-neural cortex 1 gene (ENC1), belonging to the oxidative stress pathway, was increased in asymptomatic patients compared to symptomatic patients [146]. ENC1 is a negative regulator of NRF2 by suppressing NRF2 protein translation [147]. It is also crucial in the immune system and is regulated by interferons [146].

Furthermore, an in-silico study evaluated the expression levels of 125 oxidative stress genes, including 37 pro-oxidant genes, 32 oxidative stress-responsive genes, and 56 antioxidant genes in the online available transcriptomic datasets. Nine datasets were included in the analysis. Seven oxidative stress-related genes were upregulated in whole blood samples and lung autopsies in severe cases compared to non-severe. The upregulated genes were myeloperoxidase (MPO), S100 calcium-binding protein A8 (S100A8), S100 calcium-binding protein A9 (S100A9), sulfiredoxin-1 (SRXN1), glutamate-cysteine ligase regulatory subunit (GCLM), sestrin 2 (SESN2), and thioredoxin (TXN). MPO, S100A8, and S100A9 were the topmost significantly upregulated genes in severe versus asymptomatic patients [147]. MPO is a pro-oxidative protein catalyzing ROS formation [148], whereas S100A8 and S100A9 are a part of the calprotectin complex having a tremendous effect on the net inflammation and redox balance [147].

Another in silico study evaluated one transcriptomic dataset finding out that several genes related to the cellular response to stress were dysregulated in cases of SARS-CoV-2 infection, such as NOX2 and dual oxidase 1 (DUOX1) [149]. NOX2 participates in ROS production [149]. It is also involved in regulating both innate and adaptive immunity. It mediates the function of type I interferons, the inflammasome, phagocytosis, antigen processing and presentation, and cell signaling [150]. DUOX1 contributes to ROS production and the induction of hydrogen peroxide synthesis. It is also involved in immune cells and is essential for modulating phagocyte activity and cytokine secretion [150].

An in silico study evaluating different data sets dealt with Alzheimer’s disease (AD) and COVID-19. It was observed that the estrogen receptor alpha gene (ESR1) expression was downregulated in patients with AD and COVID-19 compared to AD patients only. ESR1 has neuroprotective properties and protects the central nervous system against beta-amyloid, oxidative stress, and inflammation [151]. ESR1 was shown to be downregulated in AD, which causes its inability to subside neurotoxicity and may lead to a worse prognosis of COVID-19 in AD patients [152].

Another study evaluated one RNA sequencing dataset retrieved from COVID-19 patients and healthy controls and has enriched results for expression in dendritic and natural killer cells. They found that 15 genes were differentially expressed in dendritic cells and 44 in natural killer cells. Some of these genes are involved in oxidative stress-related pathways. Among those are metallopeptidase domain 9 (ADAM9), cathepsin B (CTSB), and RAR-related orphan receptor A (RORA) [153]. ADAM9 is induced by oxidative stress. It is related to prostate cancer cell survival and progression [154]. CTSB regulates the activation of the NOD-, LRR-, and pyrin domain-containing protein 3 (NLRP3) by hydrogen peroxide, which increases CTSB production and, consequently, the induction of the NLRP3 [155]. RORA has strong antioxidant capacities. It increases the production of antioxidant enzymes, such as SOD2 and GPX1 and decreases the production of cytokines. Agonists of RORA also reduce oxidative stress in oleic acid-induced stress in primary cultures of hepatocytes [156].

Nevertheless, another transcriptomic study revealed that ROS-related genes are enriched in severe cases of COVID-19. Differentially expressed genes from the oxidative stress-associated pathways are TXN, SRXN1, peroxiredoxin-4 (PRDX4), peroxiredoxin-1 (PRDX1), MPO, microsomal GST 1 (MGST1), late endosomal/lysosomal adaptor, MAPK and MTOR activator 5 (LAMTOR5), ferritin light chain (FTL), and cyclin-dependent kinase inhibitor 2D (CDKN2D) [157]. TXN expression has already been associated with COVID-19 in another study [147]. It has antioxidant properties and reduces influenza A virus (H1N1)-Induced acute lung injury and inflammation in the lungs of the virus-infected mice [147]. Furthermore, SRXN1 is an endogenous antioxidant protein that may alleviate cell oxidative stress damage [158]. Similarly, PRDX4, PRDX1, and MGST1 all have antioxidant capacities with their ROS scavenging abilities [159,160,161]. LAMTOR5 mediates the interaction between NRF2 and KEAP1, which regulates oxidative stress, as shown in osteosarcoma cells [162]. It has been reported that defects in the FTL cause an increase in iron levels and, thus, oxidative stress, contributing to neurodegeneration in mice. This indicates that FTL has an antioxidative role under physiological conditions [163]. On the other hand, CDKN2B seems to act as a pro-oxidative agent, as shown in hepatocellular carcinoma cells [164].

Finally, a study on hospitalized children infected with SARS-CoV-2 showed a unique monocyte/dendritic cell gene signature correlated with severe myocarditis. It was characterized by sustained nuclear factor κB (NF-κB) activity and tumor necrosis factor-alpha (TNF-α) signaling and was associated with decreased gene expression of NF-κB inhibitors. They also showed enrichment in hyper inflammation and response to oxidative stress-related genes. Expression of genes like HIF1A, HMOX1, and high mobility group box 1 (HMBG1) was associated with myocarditis in COVID-19 patients. HIF-1α is a sensor of oxidative stress. It can induce a switch from oxidative phosphorylation to glycolysis to limit ROS generation [165]. HMOX1 plays an essential role in oxidative stress defense [166], while HMBG1 is a sensitive marker of oxidative DNA damage in living cells [167].

## 9. Oxidative Stress Markers in COVID-19

Several studies evaluated the levels of various oxidative stress markers in COVID-19 patients. Thiol/disulfide misbalance is an indicator of oxidative stress [168], and it was observed in COVID-19 patients where increased levels of disulfides were found [70]. Kalem et al. found that disulfide levels are significantly higher in mild to moderate COVID-19 patients compared to control, while severe patients had similar disulfide levels compared to control [89]. Decreased thiol levels in COVID-19 patients are confirmed in several studies [66,69,71,88,89,90,92]. Decreased serum-free thiols could discriminate mild COVID-19 patients from healthy controls. Serum-free thiols are markers of oxidative stress and are usually reduced in diseases characterized by increased levels of ROS [71]. At the moment of hospitalization, levels of native thiol and total thiol were significantly lower in COVID-19 patients compared to healthy controls [69,89] and in severe compared to mild to moderate adults [88,89] and pediatric patients [88]. In hospitalized patients, thiol levels were decreasing with increasing disease severity [90], in COVID-19 patients with acute respiratory distress syndrome (ARDS) compared to those without ARDS [92], and in ICU admitted compared to non-ICU COVID-19 patients [85].

**Figure 4:**
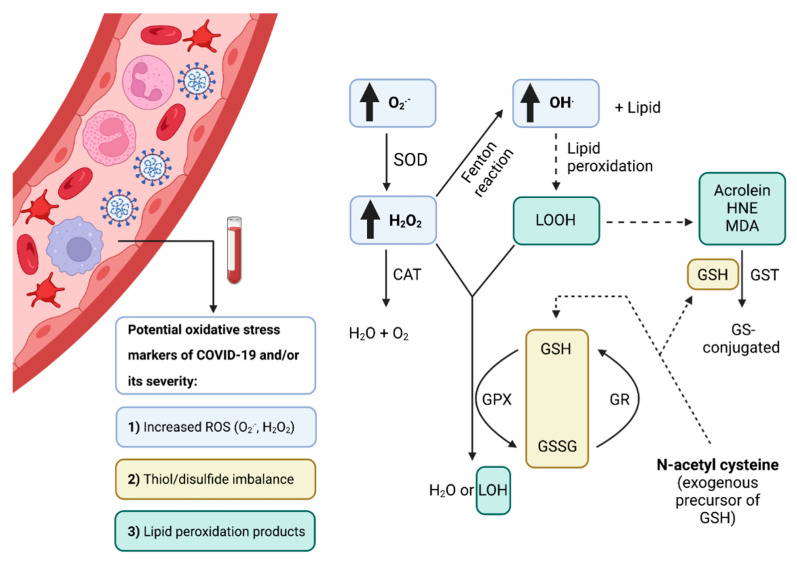
Oxidative stress biomarkers in COVID-19 development and severity. During SARS-CoV-2 infection, ROS levels are increased, and an imbalance between thiol and disulfide leads to lipid peroxidation. More specifically, O_2_^.−^ is converted to H_2_O_2_ via SOD, which is further converted into H_2_O and O_2_ through CAT. In the Fenton reaction, levels of OH^.^ are increased, which then reacts with lipids to initiate lipid peroxidation. Different lipid peroxidation products are derived from the process, ending with acrolein, HNE, and MDA. LOOH and H_2_O_2_ are reduced to lipid alcohols and water, respectively, by GPX. In this enzymatic reaction, reduced GSH is converted to glutathione disulfide, which is then regenerated with GR. Acrolein, HNE, and MDA are conjugated with glutathione via the GST enzymatic reaction. Since a lot of glutathione is used in the process, N-acetyl cysteine can be used to replenish it. Abbreviations: O_2_^.−^: superoxide, H_2_O_2_: hydrogen peroxide, OH-: hydroxide, LOOH: Lipid hydroperoxide, SOD: superoxide dismutase, CAT: catalase, GPX: glutathione peroxidase, GR: Glutathione reductase, GSH: glutathione, GSSG: glutathione disulfide (oxidized glutathione), LOH: lipid hydroperoxide, HNE: hydroxynonenal, MDA: Malondialdehyde, GTS: glutathione-S- transferase. Created with BioRender.com (accessed on 9 August 2022).

Increased oxidative cell damage markers such as increased levels of protein carbonyls and lipid peroxidation products malondialdehyde (MDA) and 4-hydroxynonenal (4-NHE) were observed in COVID-19 patients compared to controls [32]. A study comparing oxidative stress biomarkers in hospitalized versus outpatients found that both groups had higher MDA and advanced oxidation protein product levels at baseline. In contrast, levels of 8-hydroxy-2′-deoxyguanosine increased during the first seven days for both groups. Advanced oxidation protein product levels were significantly higher in hospitalized patients than in outpatients [74]. Total MDA levels were higher in COVID-19 patients compared to healthy controls, and there was also a difference depending on later admission to ICU. Patients placed in the ICU had significantly higher MDA levels [18]. Significantly increased levels of oxidized phospholipids were found in hospitalized COVID-19 patients compared to controls [68]. TBARS, as a measure of lipid peroxidation, had similar levels in all hospitalized patients, independent of disease severity [83]. Lage et al. found elevated lipid peroxidation and increased ferritin levels in COVID-19 mild, moderate, and severe patients compared to healthy controls. The same authors found higher mitochondrial superoxide levels in circulating classical monocytes from COVID-19 patients than in healthy controls [30]. Significantly higher levels of 4-HNE adduct proteins in the first three days of hospitalization were found in critical COVID-19 deceased compared to critical patients that survived. 4-HNE is a reactive aldehyde produced by lipid peroxidation [95]. The level of 2-hydroxybutyric acid was significantly increased in hospitalized COVID-19 patients compared to controls [67]. While Cekerevac et al. found that hydrogen peroxide levels were similar in all hospitalized patients, independent of disease severity [83], Badawy et al. found high levels of plasma hydrogen peroxide and damaged serum albumin can be used as predictors of mortality in COVID-19 [52]. Moreover, with increased COVID-19 severity in hospitalized patients, increased levels of ischemia-modified albumin were observed [90]. Figure 4 summarizes potential biochemical and molecular biomarkers of COVID-19 development and severity and antioxidative enzymes coded by polymorphic genes that may lead to interindividual variability in antioxidative capacity.

## 10. Antioxidant Capacity in COVID-19 Patients

Besides increased oxidative stress in COVID-19 patients, decreased antioxidant capacity was observed [34]. As discussed in other parts of this article, some of the main reasons for decreased antioxidant capacity could be the inactivation of the Nrf2 transcription factor and decrease in antioxidant proteins controlled by Nrf2 [62,63], as well as alterations in the activity of crucial antioxidant enzymes and processes such as GPX with GSH [30,31,75,76,78,79,87,94,98], and PON-1 [81].

Yaghoubi et al. found that decreased total antioxidant capacity could be used to differentiate severe ICU admitted patients from mild COVID-19 outpatients and both groups of patients from healthy controls [84]. Martín-Fernández et al. found decreased antioxidant capacity (ABTS and FRAP) in hospitalized COVID-19 patients compared to healthy controls [32], and significantly lower total antioxidant status in ICU compared to non-ICU patients were observed [85]. In a pilot study on 21 critically ill patients, total antioxidant capacity was below the detection limit [95]. However, Karkhanei et al. found opposite results where total antioxidant capacity was increased in ICU admits compared to hospitalized non-ICU COVID-19 patients with similar total antioxidant capacity to healthy controls. The sampling time in this study was at least 24 h after admission, while the maximum time of sample collection was not indicated in the article [87]. In other articles, the earlier sampling time was used: Yaghoubi et al. collected samples upon hospital admission [84], Martín-Fernández et al. at 9 am the morning after hospitalization [32], Çakırca et al. [85], and Žarković et al. [95] on the first day from hospital admission. As the levels of oxidative stress and antioxidant capacity may depend on the course of disease [82], the sampling times could have influenced the results of Karkhanei et al. [87]. Some studies found no difference in antioxidant capacities between evaluated groups. Atanasovska et al. found that severe and moderate COVID-19 hospitalized patients had similar antioxidant capacities [73], and Lage et al. found no difference in total antioxidant response and iron levels in COVID-19 mild, moderate, and severe patients compared to healthy controls [30]. Additionally, Gadotti et al. found no difference between hospitalized moderate and severe COVID-19 patients on the first day of hospitalization for hydrogen peroxide, GSH, and oxidized GSH, MDA, carbonyl, and sulfhydryl levels. Moreover, both groups had a similar total antioxidant capacity and GST activity [91]. It is important to note that studies that found no difference in antioxidant capacities between evaluated groups had a smaller number of patients (Atanasovska et al. 33 COVID-19 patients [73], Lage et al. 31 mild, 4 moderate, and 12 severe COVID-19 patients and 21 healthy control [30], Gadotti et al. 44 moderate and 33 severe COVID-19 patients [91]) compared to studies that found decreased antioxidant capacities (Yaghoubi et al. 60 milds and 60 severe COVID-19 patients and 60 controls [84], Martín-Fernández et al. 108 hospitalized COVID-19 patients (40 intubated or with fatal outcome and 68 non-intubated or without fatal outcome) and 28 controls [32], Çakırca et al. 86 hospitalized COVID-19 patients, 40 in ICU and 46 non-ICU patients [85] (Table 2). A smaller sample size could influence the results and lead to no statistically significant difference between analyzed groups.

## 11. Potentials for Therapeutic Approaches Based on Antioxidant Therapy

There are many treatment approaches and clinical trials for COVID-19 based on antiviral drugs (remdesivir, molnupiravir), single or combinational approaches of biological agents in oral, injection, or inhalation forms [169,170,171,172,173]. Corticosteroids, convalescent plasma, and immunomodulators were also widely used. Furthermore, drugs such as antimalarials were repurposed for COVID-19 treatment, although their efficacy has not yet been proven [174]. Moreover, blood stem cells, cord blood mononuclear cells, mesenchymal stem cell (MSC) based strategies [175,176,177] or targeted therapies with monoclonal antibodies [174,178,179], such as sotrovimab, adalimumab, nivolumab, clazakizumab, are being investigated in clinical trials. Scientists are trying to find new drugs or implementation ways to overcome the detrimental effects of the disease, but there is still no effective treatment.

Antioxidative agents are one of these groups of molecules. They were first successfully used in patients with pulmonary sepsis and then proposed to alleviate septic shock in COVID-19 patients due to their antioxidant and immune defense activation properties [23]. The upregulation of systemic inflammation with pulmonary dysfunction in COVID-19 is accompanied by the production of ROS and a concomitant deficiency of antioxidants. Antioxidative enzymes such as SOD, CAT, and GPX, and nonenzymatic antioxidant and antiinflammatory molecules such as vitamins A, C, D [92,180] and E, melatonin, resveratrol, GSH, NAC, silymarin, quercetin, curcumin, propolis, thymoquinone, Boswellia, and hesperidin, were suggested as the therapeutic agents against COVID-19 and some of them are being evaluated in clinical trials [181,182,183,184,185,186,187,188,189,190,191]. Vitamin therapy was not suggested to replace classical antiviral and antiinflammatory treatments for COVID-19, but it could be used as an adjuvant therapy with other pharmacological treatments [190]. A prospective, double-blinded, randomized parallel-controlled interventional clinical study evaluated the effect of antioxidant supplements in 87 hospitalized COVID-19 patients. They reported that in patients under oral dietary supplements enriched with vitamins A, E, C, Zinc, and selenium, the levels of alkaline phosphatase, IL-6, TNF-α, and MCP-1 were significantly lower than in the placebo group, concluding that oral antioxidant supplementation has a significant effect on the clinical parameters among patients with non-critical COVID-19 [191].

Another possible strategy could rely on the activation of the nuclear erythroid-related factor 2 (Nrf2) and Nrf2/antioxidant-related elements (ARE) with the treatment strategies that are based on resveratrol, sulforaphane, melatonin, and vitamin D [181,192]. Natural compounds like propolis were suggested for blocking the proinflammatory PAK1 enzyme [186], and thymoquinone is suggested for modulating the production of nitric oxide (NO) and ROS and protection against multiple organ dysfunction syndrome (MODS) [185]. Registered clinical trials on antioxidant treatments in COVID-19 are listed in Supplementary Table S5.

However, since most studies concern GSH and NAC, as potential therapeutic candidates for COVID-19, this review mainly focused on these two antioxidant agents (Table 5 and Table 6).

### 11.1. Glutathione

GSH is the most abundant physiological antioxidant in humans. It is a low-molecular-weight thiol-containing agent detoxifying both xenobiotic and endogenous compounds. Through its active thiol group, GSH can directly interact with reactive oxygen/nitrogen species as an antioxidant or indirectly serve as a cofactor for various enzymes [193,194]. Whether the reaction is enzymic or nonenzymic, conjugation with GSH is crucial in detoxification [195]. GSH’s role in detoxification is accomplished via GSTs and GPX catalyzed reactions, antioxidant defense, and regeneration of reduced thiols [196]. The intracellular GSH levels, which are in the range of millimolar concentrations, indicate the vital role of the molecule not only in detoxification but also in protein folding, regeneration of antioxidant vitamins C and E, mitochondrial function, signaling, cellular proliferation, and apoptosis. Besides, the protection of host immune cells through GSH’s antioxidant mechanism preserves the optimal functioning of the immune system. Adequate intracellular GSH levels are also required for optimal T-lymphocyte function [197].

GSH also inhibits the replication of viruses at different stages of the viral life cycle and helps the antiviral defense by decreasing the viral load and the subsequent cytokine storm. Studies have also shown that a delicate disulfide-thiol balance partially regulates viral entry and fusion in the host cell. Thus any increases in oxidative stress or depletions in the GSH reservoir, such as those that occur in aging, cigarette smoking, chronic diseases, or low GSH intake, can contribute to an increased risk of more severe disease pathogenesis and worse outcomes like in COVID-19 cases [198,199]. Additionally, SARS-CoV-2 affects oxidative homeostasis and ROS production and inhibits GSH and NRF2, which intercept ROS damage [200]. Furthermore, there is evidence that GSH and NAC can suppress the activity of variant spikes from specific strains of the SARS-CoV-2, like alpha and delta strains [201].

In COVID-19, GSH deficiency was suggested to lead to increased viral replication and oxidative damage of the lung, resulting in hyperinflammation and ARDS. The SARS-CoV-2 infection affects the metabolism of GSH in the homeostatic control of the redox and extracellular thiols [202]. Thiol levels were suggested to be associated with the severity of COVID-19, which could be a new, sensitive prognostic biomarker in COVID-19 [66]. In addition, the role of aminothiols, like GSH and its precursor, cysteinylglycine (CG), has been investigated in patients with COVID-19. Specifically, GSH total content was associated with advanced oxidation protein product level in moderate or severe symptoms. Low levels of reduced forms of CG were associated with a high risk of lung damage [203]. Moreover, drugs containing the thiol moiety, such as erdosteine, may be utilized as novel therapeutics to block NF-kB and address the cytokine storm syndrome and respiratory distress in COVID-19 pneumonia patients [204].

The limited therapeutic approaches for COVID-19, and the role of GSH deficiency in severe cases, suggested that restoration of GSH levels in these patients would be a promising approach. GSH, its precursor NAC and selenium-based natural supplements that can biologically mimic GPX, either as stand-alone or in combined therapy, can improve the host response against COVID-19 [205]. A molecular dynamic-based study showed that GSH could be the best inhibitor to overcome COVID-19 compared to the other vitamins such as tocopherol (vitamin E), thiamine (vitamin B1), pantothenic acid (vitamin B5), pyridoxine (vitamin B6), biotin (vitamin B7) [206].

GSH can be administered via oral or intravenous routes. However, oral GSH treatments are approached cautiously because GSH given orally may be degraded by digestive peptidases. Recent research indicates that GSH in liposomal or sublingual forms is more readily bioavailable and positively impacts systemic GSH levels compared to oral and intravenous routes [207].

Published experimental studies and case reports supporting GSH supplementation in COVID-19 are summarized in Table 5. An in vitro study showed that GSH has a protective role against peroxynitrite-mediated DNA damage at the site of inflammation [208]. An in vivo study on IV and nebulization forms of S-Nitrosoglutathione (GSNO) in a mice model showed that nebulized-GSNO therapy could be used and translated into an affordable treatment in ischemic events such as strokes, especially in limited healthcare infrastructure [209]. GSH treatment was also administered in two severe COVID-19 patients with Lyme disease and tick-borne co-infections, cough, and dyspnea. A trial of 2 g of PO or IV GSH was used in both patients and improved their dyspnea within 1 h of use. Repeated use of 2000 mg of PO and IV GSH effectively relieved respiratory symptoms. Thus, oral and IV GSH can be an excellent treatment alternative for regulating NF-κB signaling and treating “cytokine storm syndrome” in COVID-19 patients [210].

COVID-19 patients had severe GSH deficiency, elevated oxidative stress, and altered oxidant damage. In a clinical study that included 60 COVID-19 patients, these effects were more pronounced with older age, although they were also observed in younger COVID-19 patients [31]. A study in a mice model showed that supplementation with GSH precursor amino acid glycine combined with NAC had significant potential to treat GSH deficiency and lower oxidative stress and oxidative damage [211]. It was suggested that such supplementation might be beneficial also for COVID-19 patients [31]. Published studies supporting GSH supplementation in COVID-19 are summarized in Table 5.

**Table 5:** Published studies supporting GSH supplementation in COVID-19.

Therapeutic Agent	Objectives of the Study	Major Findings	Type of Study	References
GSH	To explore the role of GSH and other thiols in neutralizing the effect of peroxynitrite-mediated DNA damage through stable GSH-DNA adduct formation	Protective role of GSH against the PN-mediated toxic effect at the site of inflammation	In vitro	[208]
GSNO or GSNO-Nebulization	To explore IV infusion of GSNO in nebulization (diabetic stroke, hypoxia circumstances as COVID-19)	GSNO-nebulization enhanced collateral microvascular perfusion in the early hours of hypoxia	In vivo mice model	[209]
GlyNAC	To explore the GlyNAC effect on longevity in mice	GlyNAC mice lived longer than controls, improved/corrected impaired GSH synthesis, GSH deficiency, OxS, mitochondrial dysfunction, abnormal mitophagy and nutrient-sensing, and genomic-damage	In vivo mice model	[211]
Case 1: a dose of 2 g up to twice a day PO + probiotics Case 2: liposomal l- GSH + azithromycin+ hydroxychloroquine	To explore the effects of using high dose oral and/or IV GSH in the treatment of 2 patients with dyspnea secondary to COVID-19 pneumonia	Oral and IV GSH may represent a novel treatment approach for blocking NF-κB and addressing “cytokine storm syndrome” and respiratory distress in patients with COVID-19 pneumonia	Case report (2 patients)	[210]

GSH: glutathione, NAC: N-Acetylcysteine, IV: intravenous, GSNO: S-Nitrosoglutathione, GlyNAC: combination of glycine and NAC.

### 11.2. N-Acetyl Cysteine

NAC is mainly used as a mucolytic and antiviral agent. It is metabolized into cysteine in the liver by a deacetylation process. This deacetylation can reduce various free radicals by giving one electron or acts as a nucleophile by giving one or two electrons [212].

NAC is widely used as a prescription drug and dietary supplement. It was first introduced in the 1960s as a mucolytic drug depolymerizing mucin molecule. This effect was attributed to NAC’s ability to cleave disulfide cross-bridges in the glycoprotein matrix of mucous protein complexes [213,214]. Later studies indicated that NAC also improved mucociliary clearance and modulated the virulence factors of the intrabronchial bacterial flora [215], and enhanced the antioxidant/antiviral lung defenses [216]. NAC is also an immune modulating agent with its redox balance changing ability, NF-κB suppression, controlling cytokine production, and chemotactic signals [217].

The generation of free radicals by phagocytes involved in the inflammatory process and alterations of the immune response play key roles in viral infections such as the classical flu or COVID-19. This is not surprising regarding the body’s defenses because of the release of immunoactive mediators such as interleukins (ILs), interferon-gamma (IFN-γ), and the tumor necrosis factor (TNF) is required for the elimination of the virus. This cascade of events, in turn, alters the cell redox equilibrium causing a deficiency in antioxidants. Thus, any safe treatment that supports the body’s antioxidant pool may be promising in viral infections. In vitro and in vivo administration of NAC have been reported to lead to anti-inflammatory (e.g., decreased ILs, IFN- γ, and TNF alpha concentrations) and antioxidant effects in several pulmonary diseases, including viral pneumonia [218,219]. NAC has also been investigated in experimental infection by influenza A viruses [220] and in clinical trials, including influenza patients [221]. NAC reduced the symptoms and the severity of influenza.

Subsequently, NAC was reported to be an effective antioxidant leading to significant increases in plasma and alveolar GSH concentrations. This role was attributed to its thiol (-SH) groups, by which it enriches the intracellular sulfhydryl pool and acts as a precursor of reduced GSH. Under physiological conditions, the uptake of extracellular GSH requires the breakdown of GSH to amino acids, their subsequent transport into the cell, and intracellular synthesis of the tripeptide from cysteine, glutamate, and glycine. Unlike cysteine, the rate-limiting amino acid in GSH synthesis, NAC can be effectively transported across the cell membranes and has very low toxicity [221]. A computational study showed that thiol-based chemical probes could be a strategy to inhibit the SARS-CoV-2 infection by shifting the spike glycoprotein redox scaffold [222]. Therefore, NAC could be used as a potential agent in many clinical applications of pathological conditions involving oxidative stress, including acute and chronic bronchitis, acute respiratory distress syndrome (ARDS), and certain cardiovascular diseases.

Table 6 summarizes published studies using NAC as adjuvant therapy in COVID-19. It was proposed that an increase in angiotensin 2 may induce a redox imbalance in alveolar epithelium cells that results in apoptosis, increased inflammation, and impaired gas exchange. NAC was suggested to restore this problem in COVID-19 patients. This hypothesis was tested in a retrospective, two-center cohort study (42 NAC users, 40 controls) as 600 mg bid orally for 14 days. The study showed that 1200 mg/day of oral NAC administration significantly reduced the progression rates to severe respiratory failure (SRF), mechanical ventilation requirement, and mortality [223]. In addition, a combination of NAC and bromelain showed even better mucolytic and anti-inflammatory activity ex vivo in tracheal aspirate samples from COVID-19 patients compared to NAC treatment ore controls [224]. Another study evaluated the high dose of oral 600 mg NAC every eight hours. The study reported significantly reduced mortality with the higher NAC dose but no effect on the mean duration of hospitalization, admission to the ICU, or use of invasive mechanical ventilation [225].

COVID-19 is known for its negative effects on male fertility via virus division, cytotoxic effects on testicular tissue, and immunopathological effects. NAC was also suggested to improve sperm concentration and reduce ROS and the oxidation of sperm DNA. This hypothesis was tested on 200 men for 3 months with oral 600 mg/day. Results show that oral NAC consumption significantly improved sperm total motility, sperm morphology, and sperm concentration after the COVID-19 infection [226].

A few case reports also supported NAC administration in COVID-19 patients. In a study, NAC inhalation solution was tested for 2 months on a 64-year-old patient with an esophageal cancer history. This therapy was implemented after the antibiotics, antiviral and antibacterial medications, respiratory support, expectorant nebulization, and nutritional support. This study revealed that NAC inhalation lavage might have great potential to clean the airway and overcome severe pneumonia or respiratory failures like COVID-19. Nevertheless, for using NAC as a bronchoscopic lavage therapy, a study of the reasonable dosage, safety, and efficacy in a large sample is urgently required [227]. Another case report showed that the NAC administration reversed the G6PD deficiency manifesting due to the GSH depletion. This study also reported that IV administration of different NAC doses reduced CRP and ferritin levels in nine out of ten G6PD patients. NAC mechanism of action may include blockage of viral infection and the following cytokine storm [228]. A double-blind, randomized, placebo-controlled trial (14 under NAC, 16 controls) by de Alencar et al. was based on the hypothesis that NAC administration could restore redox homeostasis. NAC was administered as 21 g (300 mg/kg) IV to the patient group; however, it did not affect the evolution of the severe COVID-19 [229]. Single center clinical trial was conducted by Taher et al. on 92 patients (45 placebo, 47 NAC group) in which IV NAC treating strategy did not cause improvement [230].

**Table 6:** Published studies on NAC administration in COVID-19 patients.

Therapeutic Agent	Objectives of the Study	Number of Patients	Major Findings	Type of Study	References
NAC + standard palliative care and drugs + remdesivir + dexamethasone	Evaluate NAC effects in hospitalized COVID-19 pneumonia cases in terms of SRF progression and mortality	82 patients (42 NAC and 40 controls)	Oral NAC provided lower SRF and mortality compared to controls	Retrospective, two-center study	[223]
NAC + standard of care	Explore the potential benefits of high NAC dose in COVID-19	19208 patients (2071 NAC, 17,137 controls)	Oral NAC provided significantly lower mortality	Observational retrospective study	[225]
NAC	Explore potential effects on sperm concentrations and quality	200 men with COVID-19 history last three months 100 NAC, 100 controls)	Oral NAC consumption significantly improved sperm total motility, sperm morphology, and sperm concentration	Interventional study	[226]
NAC inhalation after treatment with antibiotics, antiviral and antibacterial medications, respiratory support, expectorant nebulization, and nutritional support	Observe NAC inhalation solution combined with routine nebulization on patient	1 patient	Refractory hypercapnia gradually improved	Case report	[227]
NAC + hydroxychloroquine + ECMO	Explore whether GSH deficiency is reversible with NAC administration	10 patients with G6PD deficiency	NAC reduced CRP and ferritin levels of G6PD deficient patients	Case report	[228]
NAC	Explore the effects of NAC on COVID-19 severity	30 patients (14 NAC, 16 controls)	No effect on the evolution of severe COVID-19	Double-blind, randomized, placebo-controlled trial	[229]
NAC	Evaluate the potential benefits of NAC in patients with COVID19-associated ARDS	92 patients (45 placebo, 47 NAC)	No improvement in patients receiving NAC	Single center clinical trial	[230]
NAC inhalation; 5% saline solution; or 8.4% sodium bicarbonate + control group (no routine inhalation)	Evaluate the effect of routine inhalation therapy on VAP in mechanically ventilated COVID-19 patients	175 patients who were treated with mechanical ventilation	Routine inhalation therapy had no effect on the incidence of bacterial or fungal VAP nor all-cause mortality, but a significant reduction of Gram-positive and MRSA VAP was observed in the treatment groups	Randomized controlled trial	[231]
IV NAC	Investigate whether IV NAC attenuates the cytokine storm	10 COVID-19 positive patients	No benefit of IV NAC	Retrospective case series	[232]

GSH: glutathione, NAC: N-Acetylcysteine, SRF: severe respiratory failure, ARDS: acute respiratory distress syndrome, G6PD: Glucose 6-phosphate dehydrogenase, ECMO: extracorporeal membrane oxygenator, VAP: ventilator-associated pneumonia, MRSA: methicillin-resistant Staphylococcus aureus, IV: intravenous.

### 11.3. Clinical Trials on Glutathione or N-Acetyl Cysteine Supplementation in COVID-19

Searching the Clinicaltrials.gov database, we identified 22 trials using NAC and 10 trials using GSH in COVID-19 patients. Six were duplicates, so the number of trials using either GSH or NAC supplementations was 25 (Supplementary Table S6). Only seven of these trials have been completed, while four are enrolling by invitation, three are recruiting, one is active and not recruiting, six are not yet recruiting, one is terminated, one has unknown status, and two have been withdrawn. The details of the studies that are either currently recruiting patients or have been completed can be found in Table 7.

Most clinical trials focused on NAC or a combination of NAC with other compounds, such as vitamins and glycine. Two studies focused on ebselen, a synthetic drug with anti-inflammatory and antioxidant activity that mimics the action of GPX [233,234]. In addition, one study evaluated the efficacy, safety, and tolerability of dendrimer N-acetyl-cysteine (OP-101) in patients with severe COVID-19. OP-101 is derived from the conjugation of NAC to the hydroxyl dendrimer, has anti-inflammatory activity, and is considered a promising therapy for COVID-19 [235]. NAC usage as an adjunct treatment is recommended in some countries like the Philippines [236].

Different formulations and application modes may have an important effect on the bioavailability, distribution, and efficacy of both GSH and NAC. In lung diseases, for example, oral administration of GSH or nebulization with water or saline was beneficial for the patients [237,238]. NAC can also be administered orally, in a nebulization form or intravenously [239]. Interestingly, both orally and intravenously, high doses of NAC were effective in patients with severe symptoms of COVID-19 [240]. Lung-targeted liposomes have also displayed an increased accumulation profile in a mice model for ARDS treatment [241]. However, more detailed studies with clinical trials to determine the efficacy of NAC stand-alone and others for increasing its bioavailability and targeted delivery are still needed [219].

It is known that anti-COVID-19 drugs may affect apical and hepatocyte transporters of the liver and can also cause liver toxicity [242]. Therefore, there is always a great demand for non-toxic or low adverse effects and more efficient drug candidates. Administration of NAC boosts the reservoir of master antioxidant GSH and supports the immune system by reducing inflammation and providing healthy liver function. Currently, there is no evidence-based role for NAC supplementation in preventing COVID-19. However, the oral administration of NAC, at 600 mg twice daily, may be suggested for preventive purposes, especially in elderly or chronic diseased people. NAC also has the potential for preventive mechanisms on endothelial function and limiting microthrombosis in severe forms of COVID-19 [27]. Although not recommended for wide use in the prevention, systemic or aerosolized NAC administration may improve outcomes in specific patients with established COVID-19 and respiratory problems. Since COVID-19 cases manifest symptoms of systemic inflammation and have thick respiratory mucus preventing adequate oxygenation, the clinical course of some patients may be complicated by ARDS. Systemic or aerosolized NAC may be beneficial in this specific patient population. The treatment is inexpensive, simple, and has no known adverse effects or typical drug interactions. GSH therapy is also suggested as stand-alone or combined therapy to reduce the oxidative stress over the cells, relieve sleeping problems, block NF-kB activation, addressing the cytokine storm syndrome.

## 12. Conclusions

Our systematic review of published literature and databases identified a growing body of data indicating that infection with SARS-CoV-2 leads to increased oxidative stress (Figure 1). It shows that the imbalance between the production of free radicals and the antioxidant systems, along with the underlying molecular and genetic mechanisms, plays an important role in the pathogenesis and severity of COVID-19 (Figure 4). However, it should be considered that most of the studies we identified and elaborated on in our review were performed in the first year of the pandemic. Given that these findings are preliminary and might be incomplete, more clinical studies and experimental data are needed to replicate the associations mentioned in this review. Oxidative stress pathways may be induced directly by SARS-CoV-2 spike protein and pro-oxidative enzymes or indirectly via activation of damage response pathways, such as inflammasomes, also resulting in activation of inflammatory pathways leading to a vicious cycle of increased oxidative and nitrosative stress. Both trigger lipid peroxidation, protein inactivation, and DNA damage leading to mitochondrial dysfunction, cell death, and increased inflammatory response. Indeed, increased oxidative damage markers were observed in COVID-19 patients compared to controls. Antioxidants such as GSH and antioxidative enzymes such as SOD, CAT, and GPX play an important role in preventing or reducing oxidative damage. However, despite higher CAT and SOD levels and activity reported in COVID-19 patients, the ROS levels in patients were increased and correlated with disease severity.

Nevertheless, despite a significant increase in the activity and level of antioxidative enzymes, decreased reduced thiol levels and decreased total antioxidant capacity were observed in COVID-19 patients and correlated with disease severity and worse disease outcome. GSH plays a vital role in defense against oxidative stress, both as a cofactor of GPX) and as a compound with a thiol group capable of directly reducing reactive species. NAC is an important donor for the regeneration of GSH and has antioxidant activity (Figure 2). Therefore, several preclinical studies and clinical trials were conducted and are ongoing to see if, among other antioxidative therapies, supplementation with GSH, NAC, their precursors, or other thiols could be used to improve outcomes in COVID-19 treatment.

## Figures and Tables

**Table 1:** Overview of COVID-19 case-control studies related to oxidative stress.

Patients	Major Findings	References
430 hospitalized patients and 173 controls	Decreased thiol levels in patients versus controls	[66]
Serum from 263 hospitalized patients and 280 controls	Increased Level of 2-hydroxybutyric acid in patients versus controls	[67]
182 hospitalized patients and 91 controls	Increased soluble Nox2-derived peptide in patients versus controls and ICU patients compared to non-ICU patients	[58]
108 hospitalized patients and 28 controls	Increased levels of SOD, CAT, oxidative cell damage (protein carbonyls and lipid peroxidation products MDA and 4-HNE), and decreased antioxidant capacity (ABTS and FRAP) in patients versus controls	[32]
72 hospitalized patients and 10 controls	Increased oxidized phospholipids in patients versus controls	[68]
50 hospitalized patients and 43 controls	Increased nitric oxygen levels and decreased native thiol and total thiol levels in patients versus controls	[69]
60 hospitalized patients and 24 controls	Increased lipid peroxidation and damage due to oxidative stress and decreased GSH in patients versus controls	[31]
52 patients and 34 controls	Increased disulfide, disulfide/native thiol ratio, and disulfide/total thiol ratio; decreased meteorin-like protein, native thiol, and native thiol/total thiol ratio in patients versus controls and no difference in total thiol levels	[70]
29 patients and 30 controls	Decreased serum-free thiols in patients versus controls	[71]
25 hospitalized patients and 25 controls	Increased prooxidant-antioxidant balance in patients versus controls	[72]
33 hospitalized patients	Increased oxidative stress (levels of peroxides in plasma and oxidative stress index); decreased Vitamin D and no differences in antioxidant capacity in more severe COVID-19 cases	[73]
40 pediatric patients and 35 healthy children	Increased total oxidant status and oxidative stress index and decreased Nrf2 and total antioxidant status in patients versus controls	[63]
58 patients (42 hospitalized and 16 outpatients)	Advanced oxidation protein product levels are significantly higher in hospitalized patients	[74]
Hospitalized patients (14 not admitted to ICU and 10 admitted to ICU) and 24 controls	Increased total oxidant status, CAT and SOD activity, and total MDA level in patients versus controls; Total MDA level and SOD activity at admission to hospital were higher in patients that were later placed in ICU	[18]
10 mild to moderate outpatients (4–7 weeks after infection) and 15 controls	Decreased mitochondrial function in platelets and concentration of coenzyme Q10 in blood and platelets in patients versus controls	[48]
Seminal fluid from 84 hospitalized male patients and 105 male controls	Increased ROS levels and SOD activity in patients versus controls	[75]
Placentas from 12 asymptomatic mothers, 18 symptomatic and 16 controls	Increased DNA oxidative damage and decreased CAT and GSS activity in placentas of patients versus controls; a trend of decreased SOD1 and GSR activities was observed but without statistical significance	[76]
Autopsy brain tissues from 10 patients and 10 controls	Increased oxidative stress with increased GSSG/GSH ratio in patients versus controls	[77]
Post-mortem cerebral cortex samples from 3 patients and 3 controls	Decreased GSH in patients versus controls	[78]
Post-mortem testis tissue samples from 6 patients and 6 controls	Decreased GSH in patients versus controls	[79]
Post-mortem testes and epididymis samples from 10 patients who died due to COVID-19 and 7 controls	Increased oxidative stress damage in patients versus controls	[80]
Post-mortem samples from 15 patients who died due to COVID-19	Increased nitrosative stress in patients versus controls; SARS-CoV-2 infection-induced changes in mitochondrial structure and function	[49]
126 hospitalized COVID-19 patients, 45 hospitalized patients negative for SARS-CoV-2, 50 controls	Decreased PON1 activity in COVID-19 patients versus controls	[81]

All studies in the table are of prospective design except the last one, which was a retrospective study. Prospective studies were sorted based on the number and characteristics of subjects included in the study and the types of samples analyzed. Abbreviations: SOD: superoxide dismutase, CAT: catalase, MDA: Malondialdehyde, 4-HNE: 4-Hydroxynonenal, GSSG: glutathione disulfide, GSH: glutathione, ICU: intensive care unit, RAGE: Receptor for advanced glycation endproducts, HMGB1: high mobility group box-1 protein, COX2: cyclooxygenase-2, NOX4: nicotinamide adenine dinucleotide phosphate (NADPH) oxidase 4, coQ10: coenzyme Q10, IMA: ischemia-modified albumin, PBMC: peripheral blood mononuclear cell, Nrf2: nuclear factor erythroid 2–related factor 2, PON1: paraoxonase-1.

**Table 2:** Overview of studies that focused on COVID-19 severity.

Patients	Outcome	References
144 hospitalized patients (117 mild to moderate and 27 severe) and 70 controls	Increased disulfide levels in mild to moderate patients compared to controls; decreased native and total thiol levels in patients versus controls and severe compared to mild to moderate patients; no difference in disulfide levels in severe patients compared to controls	[89]
60 mild, 60 severe patients and 60 controls	Decreased total antioxidant capacity in patients compared to controls and in severe compared to mild COVID-19 patients; no difference in nitric oxide levels, and serum activities of SOD and CAT	[84]
160 hospitalized patients (31 mild, 36 moderate, 36 severe, 57 critical)	With the increased severity of the disease, increased levels of IMA were observed. With increased severity of disease, decreased levels of thiols were observed	[90]
86 hospitalized patients (46 children, 40 adults) and 67 controls (33 children, 34 adults)	No difference in total antioxidant status and total oxidant status	[88]
127 hospitalized patients (17 mild, 40 moderate and 70 severe)	Levels of superoxide anion significantly increase with increased disease severity. CAT activity in severe COVID-19 compared to moderate and mild cases. However, moderate forms had significantly lower CAT activity compared to mild forms. Decreased nitric oxide levels in severe compared to mild and moderate COVID-19 and no difference in TBARS as a measure of lipid peroxidation, hydrogen peroxide levels, SOD, and GSH activity	[83]
59 patients (19 mild to moderate, 23 ICU admitted and 17 reporting COVID-19 history) and 34 controls	Increased RAGE, HMGB1, and COX2 in patients admitted to the ICU compared to controls	[33]
96 hospitalized patients (35 non-ICU, 19 ICU with endotracheal intubation, 24 ICU without endotracheal intubation) and 18 controls	Increased total antioxidant capacity in ICU compared to non-ICU patients and association of total oxidant status with increased severity of COVID-19. Decreased GSH levels with increased COVID-19 severity. No difference in total antioxidant capacity in non-ICU patients compared to controls	[87]
86 hospitalized patients (40 ICU and 46 non-ICU)	Increased total oxidant status and oxidative stress index and decreased thiol levels and total antioxidant status in ICU patients	[85]
77 hospitalized patients (44 moderate and 33 severe)	No difference in total antioxidant capacity, GST activity, H_2_O_2_ levels, GSH, oxidized GSH, MDA, carbonyls, sulfhydryl’s	[91]
60 hospitalized patients (29 with ARDS and 31 without ARDS)	Decreased total and native thiol levels in severe compared to mild/moderate patients in both pediatric and adult populations	[92]
58 hospitalized patients (35 non-severe, 23 severe) and 30 controls	Increased coenzyme Q10, MDA, NOX4, and IMA and levels of reduced Q10 in patients versus controls; In severe compared to non-severe COVID-19 patients, increased Q10, MDA, and ischemia-modified albumin were observed while levels of reduced Q10 were similar	[93]
31 mild, 4 moderate and 12 severe patients and 21 controls	Increased lipid peroxidation, CAT and SOD activity, ferritin and mitochondrial superoxide in circulating monocytes, and decreased GSH levels in PBMC lysates of patients versus controls; no difference in total antioxidant response and iron levels; inflammasome activation, loss of mitochondrial membrane potential, and metabolic shift from OXPHOS to glycolysis in patients with different severity; these changes were still observed after short term recovery, independently of disease severity	[30]
50 hospitalized patients (20 moderate, 30 severe)	Increased oxidative stress in patients with fatal outcomes was observed after 7 hospitalization days, while in surviving patients, oxidative stress was decreased	[82]
39 patients with critical COVID-19	Increased plasma H_2_O_2_ and damaged serum albumin in patients with fatal outcome	[52]
34 hospitalized patients (34 mild, 22 moderate to severe)	Decreased GSH in moderate and severe patients compared to mild	[94]
31 hospitalized patients admitted to ICU	No relation between oxidative stress markers’ levels at admission to ICU and COVID-19 outcome	[86]
21 critically ill COVID-19 patient (14 recovered and 7 fatal outcome)	HNE adduct proteins in the first three hospitalization days were decreased compared to surviving patients; Total antioxidant capacity was below the detection limit	[95]
Nine critically ill hospitalized patients	Systemic oxidative stress strongly altered in critically ill COVID-19 patients as evidenced by increased lipid peroxidation and deficits in vitamin C, glutathione, thiol proteins, and selenium	[96]

All studies in the table are of prospective design. Studies were sorted based on the number and characteristics of subjects included in the study. Abbreviations: SOD: superoxide dismutase, CAT: catalase, TBARS: thiobarbituric acid reactive substances, GSH: glutathione, GST: glutathione-S-transferase, MDA: malondialdehyde, H_2_O_2_: hydrogen peroxide, IMA: ischemia-modified albumin, ICU: intensive care unit, ARDS: acute respiratory distress syndrome, HNE: 4-hydroxynonenal.

**Table 3:** Overview of candidate gene studies that involved genes and genetic variants in oxidative stress pathways in COVID-19 patients.

Genes and Variants	Genotyping Method	Cohort	Origin	Outcome	References
*GSTM1* and *GSTT1* deletions; *GSTP1* rs1138272, rs1695; *GSTA1* rs3957357; *GSTM3* rs1332018	Multiple PCR (deletions), PCR-RFLP	207 patients, 252 controls	Serbian, Caucasian	Association between rs1695 and rs1332018 heterozygotes and rs1138272 Val allele carriers with decreased COVID-19 risk	[103]
*GSTM1* and *GSTT1* deletions	Multiple PCR	269 patients (149 mild and 120 severe)	NA	Association between *GSTM1*+/+ and *GSTT1*−/− genotypes and poor survival rate	[104]
*Nrf2* rs6721961, *SOD2* rs4880, *GPX1* rs1050450, *GPX3* rs8177412, and *GSTP1* (rs1695 and rs1138272) haplotype	PCR-RFLP, real-time PCR, 2-pair primers (CTPP) PCR	229 patients and 229 controls	Serbian, Caucasian	Association between GSTP1 haplotype and COVID-19 risk. The association of the SOD2*Val allele with increased levels of fibrinogen and ferritin and the association between the GPX1*Leu allele was associated with D-dimmer	[105]
*MBL2* rs1800450; *NOS3* rs1799983, intron 4 23bp VNTR	PCR-RFLP	79 patients, 100 controls	NA	No statistically significant difference neither in genotypic nor allelic level	[106]

NA: not available.

**Table 4:** Overview of GWAS studies in COVID-19.

Type of Research	Number of Participants	Ethnicity	Number of Associations	References
Meta-analysis	835 patients and 1255 controls from Italy and 775 patients and 950 controls from Spain	European	25	[129]
Meta-analysis	1457 genotyped (598/859 with severe/mild symptoms) and 1141 sequenced (severe/mild: 474/667) Chinese patients. Further incorporated 1401 genotyped and 948 sequenced ancestry-matched population controls. Genome-wide association was tested on 1072 severe cases versus 3875 mild or population controls, followed by a trans-ethnic meta-analysis with summary statistics of 3199 hospitalized cases and 897,488 population controls from the COVID-19 Host Genetics Initiative	Chinese	0	[130]
Original research	1723 outpatients with at least one risk factor for disease severity from the COLCORONA clinical trial	European	3	[132]
Original research	2244 critically ill patients with COVID-19 from 208 UK ICUs	Mixed	8	[133]
Original research	1778 infected cases	European	12	[134]
Meta-analysis	3288 COVID-19 patients676,840 controls	European	3	[135]
Meta-analysis	1,051,032 23 andMe research participants	Mixed	360	[136]
Meta-analysis	1678 COVID-19 cases and 674,635 controls	NA	2	[137]
Initiative	105 studies	Global initiative	0	[139]
Original research	175,977 participants	European	17	[138]
Original research	COVID-19 phenotypes: 482 hospitalized and 164 non-hospitalized participants	GWAS: Arabic trans-ancestry meta-analysis: European, American, South Asian, and East Asian	8	[131]

ICU: intensive care unit, COLCORONA: Colchicine Coronavirus SARS-CoV2 Trial, NA: not available.

**Table 7:** Registered clinical trials on NAC or GSH supplementation in COVID-19 treatment that are recruiting patients or have been completed.

Clinical Trial ID Number	Title	Location	Interventions	Status	Study Results
NCT04703036	Glutathione, Oxidative Stress and Mitochondrial Function in COVID-19	United States	Glycine, NAC, alanine	Recruiting	No Results Available
NCT04458298	A Study to Evaluate OP-101 (Dendrimer N-acetyl-cysteine) in Severe Coronavirus Disease 2019 (COVID-19) Patients	United States	OP-101, placebo	Recruiting	No Results Available
NCT04573153	Metabolic Cofactor Supplementation and Hydroxychloroquine Combination in COVID-19 Patients	Turkey	Hydroxychloroquine + metabolic cofactor, hydroxychloroquine + sorbitol	Recruiting	No Results Available
NCT04483973	SPI-1005 Treatment in Severe COVID-19 Patients	United States	Ebselen, placebo	Enrolling by invitation	No Results Available
NCT04484025	SPI-1005 Treatment in Moderate COVID-19 Patients	United States	Ebselen, placebo	Enrolling by invitation	No Results Available
NCT04797871	Resistance Training and Clinical Status in Patients With Post Discharge Symptoms After COVID-19	Spain	Behavioral: Resistance training, Standard care	Enrolling by invitation	No Results Available
NCT04569851	Clinical Characteristics and Prognostic Factors of Patients With COVID-19 (Coronavirus Disease 2019)	Spain	NA	Enrolling by invitation	No Results Available
NCT04742725	A Study to Evaluate the Efficacy and Safety of Prothione Capsules for Mild to Moderate COVID-19	Rwanda	Prothione, placebo	Completed	No Results Available
NCT04792021	Effect of N-acetylcysteine on Oxidative Stress in COVID-19 Patients	Egypt	NAC	Completed	No Results Available
NCT04419025	Efficacy of N-Acetylcysteine (NAC) in Preventing COVID-19 From Progressing to Severe Disease	United States	NAC	Completed	No Results Available
NCT04900129	Inhalation of Vapor With Medication (Diclofenac Sodium, Menthol, Methyl Salicylate and N-Acetyl Cysteine) Reduces Oxygen Need and Hospital Stay in COVID-19 Patients—A Case Control Study	Bangladesh	Combination of menthol, methyl salicylate, NAC and diclofenac sodium	Completed	No Results Available
NCT04570254	Antioxidants as Adjuvant Therapy to Standard Therapy in Patients With COVID-19	Mexico	Vitamin C, vitamin E, melatonin, NAC, pentoxifylline	Completed	No Results Available
NCT04755972	Mucolytics in Patients on Invasive Mechanical Ventilation Due to Severe Acute Respiratory Syndrome Coronavirus 2	Croatia	Inhalation of NAC, inhalation of sodium chloride, inhalation of sodium bicarbonate	Completed	No Results Available
NCT04666753	Retrospective Study of ImmunoFormulation for COVID-19	Spain	ImmunoFormulation	Completed	No Results Available

NAC: N-Acetylcysteine, NA: not available.

## Supplementary Materials

The following supporting information can be downloaded at: https://www.mdpi.com/article/10.3390/antiox11081609/s1: Supplementary File S1: Supplementary Methods; Supplementary Table S1: Genes identified in GWAS studies in COVID-19; Supplementary Table S2: Output of the pathway enrichment analysis for the GO biological process; Supplementary Table S3: Output of the pathway enrichment analysis using the Kyoto Encyclopedia of Genes and Genomes database; Supplementary Table S4: Output of the pathway enrichment analysis for the Reactome database; Supplementary Table S5: Identified registered clinical trials on antioxidants in COVID-19; Supplementary Table S6: Identified registered clinical trials on GSH or NAC supplementation in COVID-19. References [243,244,245,246,247,248,249,250,251] are cited in the supplementary materials.
