# Supplementary material for: Molecular Mechanisms Related to Responses to Oxidative Stress and Antioxidative Therapies in COVID-19: A Systematic Review
Source: Antioxidants (Basel). 2022 Aug 19;11(8):1609. doi: 10.3390/antiox11081609 (PMC9405444; doi:10.3390/antiox11081609)
Supplement: Supplementary file 1 [file antioxidants-11-01609-s001.zip › antioxidants-1806454-supplementary/COVID oxidative stress_Supplementary material_final_v2.pdf]

Supplementary material

Methods

To identify the role of oxidative stress in COVID-19, we systematically searched the PubMed database, using the term (COVID-19 or SARS-CoV-2) and ("oxidative stress"). Our search identified 730 articles. After removing duplicates, 197 were selected based on the title and out of them, 56 were selected based on the abstract read. Finally, 26 case-control studies and 16 studies related to COVID-19 severity were included in the review.

To identify the genetic association studies focusing on genetic polymorphisms in antioxidative enzymes and oxidative stress-related pathways in COVID-19, we narrowed the search of the PubMed database by adding the terms (genetics or genomics or genes or polymorphism or "genetic variations"). We found 142 records, of which 14 reports were included in the review. Additional articles were also identified and included from the studies' references.

To identify transcriptomic studies, we narrowed the search of the PubMed database by adding the keyword (transcriptomics) and received 29 hits. We examined the articles in the list and identified seven studies reporting a transcriptomic analysis in COVID-19 patient clinical samples with results related to oxidative stress.

We have also searched the GWAS catalog for GWAS studies in COVID-19 patients. We have identified 11 studies and have included them in the pathway enrichment analysis. First, we checked the gene names in the HUGO Gene Nomenclature Committee (HGNC) database to ensure that the names listed in the original articles were the ap-proved symbols [1]. Then we used the DAVID functional annotation tool to determine whether genes from the provided gene list could be assigned to any of the pathways involved in oxidative stress and oxidative stress response [2,3]. The analysis was performed using databases, such as GO (Gene ontology) biological process [4,5], Kyoto Encyclopedia of Genes and Genomes (KEGG) [6–8], and Reactome [9].

Regarding the antioxidative therapies currently evaluated in the clinical trials for COVID-19 treatment, we systematically searched the PubMed database and the Clini-caltrials.gov website. For the PubMed search, we used as keywords (SARS CoV-2 OR COVID-19) (n-acetylcysteine OR NAC) (ther* OR treat*) and (SARS CoV-2 OR COVID-19) (glutathione OR GSH) (ther* OR treat*) and for the clinical trials we searched on condition or disease as COVID-19 with keywords "N-Acetylcysteine" and "Glutathione". PubMed records were 121 for (glutathione OR GSH) and 54 for (N-Acetylcysteine OR NAC). All emerged records were screened manually, and duplicates and non-English publications were excluded. After removing duplicated and non-English publications, 119 and 53 respective records were retrieved and checked for the following eligibility criteria: availability of the full-texts and preclinical or clinical studies that used N-Acetylcysteine (NAC) or glutathione (GSH) as mono- or combination therapy in experimental design setting or COVID-19 patients. Studies that focused on the potential role of NAC or GSH in the treatment of COVID-19 were also considered eligible for inclusion in the review. Finally, four PubMed records related to GSH treatment and seven related to NAC treatment were included in the review.

In addition, we have searched the Clinicaltrials.gov website using the search term "COVID-19" as a condition or disease in combination with "Antioxidant" as the other term and identified 132 records. To narrow the search down to specific therapies, "Glutathione" and "N-Acetylcysteine" were used as the other term, leading to the identification of 10 and 22 clinical trials, respectively. However, many trials overlapped between the hits of both searches. After removing duplicates and studies with unknown status, suspended, ter-minated, withdrawn, or were not recruiting yet, 14 ongoing or completed clinical trials remained for inclusion in the review.

References

1. Tweedie, S.; Braschi, B.; Gray, K.; Jones, T.E.M.; Seal, R.L.; Yates, B.; Bruford, E.A. Genenames.org: The HGNC and VGNC resources in 2021. *Nucleic Acids Res.* **2021**, *49*, D939–D946, doi:10.1093/NAR/GKAA980.
2. Huang, D.W.; Sherman, B.T.; Lempicki, R.A. Bioinformatics enrichment tools: paths toward the

comprehensive functional analysis of large gene lists. *Nucleic Acids Res.* **2009**, *37*, 1–13, doi:10.1093/NAR/GKN923.
3. Huang, D.W.; Sherman, B.T.; Lempicki, R.A. Systematic and integrative analysis of large gene lists using DAVID bioinformatics resources. *Nat. Protoc.* **2009**, *4*, 44–57, doi:10.1038/NPROT.2008.211.
4. Carbon, S.; Douglass, E.; Good, B.M.; Unni, D.R.; Harris, N.L.; Mungall, C.J.; Basu, S.; Chisholm, R.L.; Dodson, R.J.; Hartline, E.; et al. The Gene Ontology resource: enriching a GOld mine. *Nucleic Acids Res.* **2021**, *49*, D325, doi:10.1093/NAR/GKAA1113.
5. Ashburner, M.; Ball, C.A.; Blake, J.A.; Botstein, D.; Butler, H.; Cherry, J.M.; Davis, A.P.; Dolinski, K.; Dwight, S.S.; Eppig, J.T.; et al. Gene Ontology: tool for the unification of biology. *Nat. Genet.* **2000**, *25*, 25, doi:10.1038/75556.
6. Kanehisa, M.; Furumichi, M.; Sato, Y.; Ishiguro-Watanabe, M.; Tanabe, M. KEGG: integrating viruses and cellular organisms. *Nucleic Acids Res.* **2021**, *49*, D545–D551, doi:10.1093/NAR/GKAA970.
7. Kanehisa, M.; Goto, S. KEGG: kyoto encyclopedia of genes and genomes. *Nucleic Acids Res.* **2000**, *28*, 27–30, doi:10.1093/NAR/28.1.27.
8. Kanehisa, M. Toward understanding the origin and evolution of cellular organisms. *Protein Sci.* **2019**, *28*, 1947–1951, doi:10.1002/PRO.3715.
9. Jassal, B.; Matthews, L.; Viteri, G.; Gong, C.; Lorente, P.; Fabregat, A.; Sidiropoulos, K.; Cook, J.; Gillespie, M.; Haw, R.; et al. The reactome pathway knowledgebase. *Nucleic Acids Res.* **2020**, *48*, D498–D503, doi:10.1093/NAR/GKZ1031.
